# Condensin II protein dysfunction impacts mitochondrial respiration and mitochondrial oxidative stress responses

**DOI:** 10.1242/jcs.233783

**Published:** 2019-11-20

**Authors:** Emily Deutschman, Jacqueline R. Ward, Avinash Kumar, Greeshma Ray, Nicole Welch, Madeleine E. Lemieux, Srinivisan Dasarathy, Michelle S. Longworth

**Affiliations:** 1 Department of Inflammation and Immunity, Cleveland Clinic Lerner Research Institute, Cleveland, OH 44195, USA; 2 Department of Genetics and Genome Sciences, Case Western Reserve University Cleveland, OH 44106, USA; 3 Bioinfo, Plantagenet, ON K0B 1L0, Canada

**Keywords:** Condensin, Mitochondria, Oxidative stress

## Abstract

The maintenance of mitochondrial respiratory function and homeostasis is essential to human health. Here, we identify condensin II subunits as novel regulators of mitochondrial respiration and mitochondrial stress responses. Condensin II is present in the nucleus and cytoplasm. While the effects of condensin II depletion on nuclear genome organization are well studied, the effects on essential cytoplasmic and metabolic processes are not as well understood. Excitingly, we observe that condensin II chromosome-associated protein (CAP) subunits individually localize to different regions of mitochondria, suggesting possible mitochondrial-specific functions independent from those mediated by the canonical condensin II holocomplex. Changes in cellular ATP levels and mitochondrial respiration are observed in condensin II CAP subunit-deficient cells. Surprisingly, we find that loss of NCAPD3 also sensitizes cells to oxidative stress. Together, these studies identify new, and possibly independent, roles for condensin II CAP subunits in preventing mitochondrial damage and dysfunction. These findings reveal a new area of condensin protein research that could contribute to the identification of targets to treat diseases where aberrant function of condensin II proteins is implicated.

## Abstract (precis)

**Highlighted Article:** Condensin II proteins localize to mitochondria, and their depletion results in defects in oxygen consumption rates, changes in ATP levels and inefficient mitochondrial responses to oxidative stress.

## INTRODUCTION

The condensin II complex, which is found in the nucleus and in the cytoplasm throughout the cell cycle, regulates DNA organization (Hirano, 2012, 2016; Kalitsis et al., 2017; Ono et al., 2004; Li et al., 2015a; Rowley et al., 2019; Bauer et al., 2012; Hartl et al., 2008; Joyce et al., 2012; Rosin et al., 2018) and maintains genome stability (Woodward et al., 2016). Our lab and others have also shown that loss of condensin II subunits can lead to aberrant regulation of gene expression (Li et al., 2015a,b; Longworth et al., 2012; Nair et al., 2019; Deutschman et al., 2018; Schuster et al., 2013; Klebanow et al., 2016; Schuster et al., 2015; Rawlings et al., 2011; Dowen et al., 2013; Yuen et al., 2017; Xu et al., 2006). In most higher eukaryotes, the condensin II complex consists of two structural maintenance of chromosome (SMC) subunits, SMC2 and SMC4, that heterodimerize to form an active ATPase needed to constrain positive supercoils (Nasmyth and Haering, 2005). The complex also consists of chromosome-associated protein (CAP) subunits including the kleisin NCAPH2, and the α-helical Huntingtin, elongation factor 3, PR65/A and TOR (HEAT)-repeat containing proteins, NCAPD3 and NCAPG2 (Nasmyth and Haering, 2005). Phosphorylation of the CAP proteins promotes complex assembly and DNA association (Abe et al., 2011; Bakhrebah et al., 2015; Kagami et al., 2017). In addition to its nuclear localization and functions, condensin II has also been reported to be present in the cytosol (Ono et al., 2004), suggesting possible secondary functions for the complex outside of the nucleus. However, the cytoplasmic organelles to which condensin II localizes, and the impacts of condensin II-mediated regulation of functions within those organelles have yet to be discovered.

Several lines of evidence have suggested that condensin II subunits could localize to mitochondria. Previously, our laboratory identified many novel, potential binding partners of NCAPD3 in human cells (Ward et al., 2017). Over 30% of the proteins that co-precipitated with NCAPD3 in these studies were proteins known to affect mitochondrial function. Mitochondria are the main source of cellular energy, in the form of ATP, thus making them essential for cellular homeostasis and organismal viability. In mitochondria, the transport of electrons creates a gradient to drive the conversion of ADP into ATP in a process known as oxidative phosphorylation (Hatefi, 2003; Papa et al., 2012). The electron transport chain (ETC), which is found embedded in the inner mitochondrial membrane (Kühlbrandt et al., 2015), is the machinery that facilitates the process of oxidative phosphorylation (Taanman, 1999; Gilkerson et al., 2013). While the outer mitochondrial membrane encapsulates the organelle and acts in a similar manner to a cell membrane (Gellerich et al., 2000), it also possesses machinery for a number of processes including fatty acid elongation (Howard, 1970) and Ca^2+^ signaling (Rizzuto et al., 2012). In humans, the circular mitochondrial genome, which is housed within the mitochondrial matrix, encodes for 13 ETC subunits, two rRNAs and 22 tRNAs, and is packaged into protein–DNA structures termed nucleoids (Taanman, 1999; Shadel, 2008). Interestingly, bacteria, which are thought to be ancestors of mitochondria, also organize their genomes into nucleoids and possess SMC-like complexes called MukB complexes, which are reminiscent of eukaryotic condensins. MukB complexes regulate bacterial nucleoid condensation, area and segregation (Nolivos and Sherratt, 2014; Kumar et al., 2017). Additionally, bacterial nucleoid-associated proteins, like MukB, have been shown to regulate gene expression (Dillon and Dorman, 2010).

Here, we show that human condensin II subunits, NCAPD3, NCAPH2 and SMC2 do, in fact, localize to mitochondria, while the NCAPG2 subunit does not appear to associate with the organelle. Surprisingly, we observe that NCAPD3 localizes to the exterior surface of mitochondria, while NCAPH2 and SMC2 localize to the mitochondrial interior and possibly also to the outside. NCAPD3-depleted cells exhibit elevated mitochondrial respiratory function and decreases in cellular ATP levels, which occurr independently of changes in mitochondrial mass or glycolysis. Depletion of both NCAPH2 and NCAPG2 also results in decreased ATP levels. Contrary to NCAPD3 depletion, however, loss of NCAPH2 and NCAPG2 results in decreased mitochondrial respiration. Additionally, we find that NCAPD3 is necessary for the ability of mitochondria to respond to oxidative stress; however, NCAPH2 is not. Taken together, our data reveal the impact that decreases in condensin II subunit expression have on the regulation of cellular respiration and on the mitochondrial response to oxidative stress, providing novel links between condensin II protein function and cellular metabolism. Furthermore, our data suggest that condensin II subunits may be acting independently of the canonical condensin II holocomplex to regulate these important mitochondrial processes.

## RESULTS

### Condensin II subunits localize to mitochondria in human cells

**Fig. 1.**
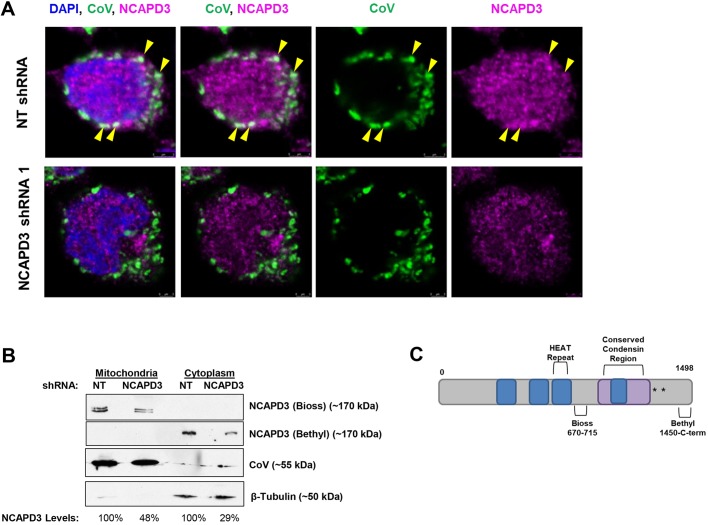
**NCAPD3 localizes to mitochondria in human cells.** (A) Immunofluorescence to detect NCAPD3 was performed in human HT-29 cells expressing NT shRNA (top row) or NCAPD3 shRNA (bottom row). DAPI is shown in blue; staining for complex V, labeling mitochondria, is shown in green, and that for NCAPD3 is shown in magenta. Yellow arrowheads point out a few examples of colocalization between NCAPD3 and complex V. (B) Equal amounts of mitochondrial and cytoplasmic lysates were isolated from equal numbers of NT and NCAPD3 shRNA-1-expressing HT-29 cells immunoblotted with antibodies targeting internal residues of NCAPD3 (Bioss, 670-715) and C-terminal residues (Bethyl, 1450-1498) of NCAPD3. Immunoblotting with antibodies against complex V and β-tubulin are shown to confirm the identity of mitochondrial and cytoplasmic fractions, respectively. NCAPD3 band intensities were normalized to the respective loading controls. NCAPD3 levels in isolated fractions from NCAPD3 shRNA-1-expressing cells were compared to levels in NT shRNA fractions, which were set to 100%. A representative of two independent experiments is shown. (C) Diagram of NCAPD3, showing protein regions detected by the respective antibodies. Blue boxes are representative of predicted HEAT repeats, the purple box represents a conserved condensin domain, and the asterisks denote experimentally identified phosphorylation sites (Abe et al., 2011; Beausoleil et al., 2004).

To test whether condensin II might localize to mitochondria in human cells, we first performed immunofluorescence analyses using primary antibodies to detect the α-subunit of complex V (ATP synthase; denoted CoV) and NCAPD3 in human colon adenocarcinoma (HT-29) cells induced to express non-targeting (NT) control short hairpin RNA (shRNA) or NCAPD3-specific shRNA (Fig. 1A) (Schuster et al., 2015). Co-staining of NCAPD3 and CoV demonstrated that NCAPD3 does, indeed, localize to mitochondria. Results also demonstrated a marked decrease in NCAPD3 immunostaining of mitochondria in NCAPD3 shRNA-expressing cells, as compared to NT shRNA-expressing cells (Fig. 1A). Immunofluorescence analysis with an antibody against *Drosophila* (d)CAP-D3 (Longworth et al., 2008) was also performed in *Drosophila* salivary glands from transgenic larvae expressing enhanced yellow fluorescent protein (EYFP) with an engineered mitochondrial localization sequence driven by the ubiquitous *spaghetti squash* promoter (*sqh-EYFP-mito*) (LaJeunesse et al., 2004). Results showed that dCAP-D3 also localizes to mitochondria in *Drosophila* (Fig. S1).

To confirm NCAPD3 localization at mitochondria, we isolated mitochondrial and cytoplasmic lysates from HT-29 cells. Interestingly, an antibody directed against internal residues of NCAPD3-detected NCAPD3 protein in mitochondrial lysate of NT shRNA-expressing cells (Fig. 1B,C), and this signal decreased in mitochondrial lysate from cells expressing NCAPD3 shRNA, suggesting that the detected protein species was, in fact, NCAPD3. Additionally, this antibody detected a NCAPD3 doublet, suggesting a modified form of the protein may also be present in mitochondria. Surprisingly, this antibody did not detect NCAPD3 in the cytosolic fraction. Conversely, an antibody targeting C-terminal residues of NCAPD3 did not detect the protein species present in mitochondria, but did detect the cytoplasmic NCAPD3 species in NT shRNA-expressing cells (Fig. 1B,C). Decreased levels of cytoplasmic NCAPD3 were also observed in NCAPD3 shRNA-expressing cells.

**Fig. 2.**
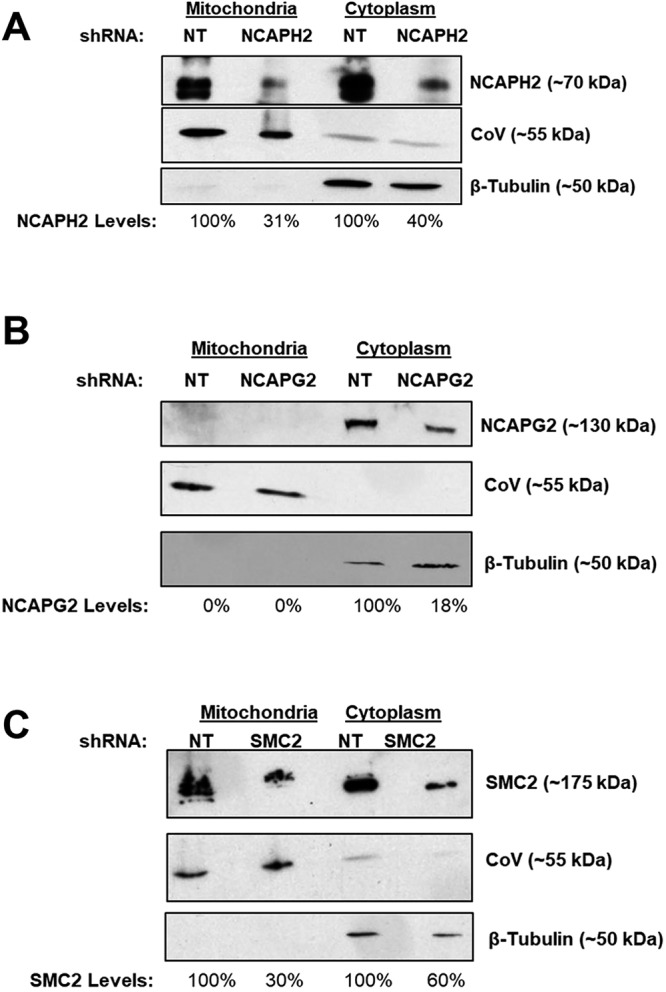
**NCAPH2 and SMC2 localize to mitochondria in human cells, while NCAPG2 does not.** (A) Equal amounts of mitochondrial and cytoplasmic lysates were isolated from equal numbers of NT and NCAPH2 shRNA-expressing cells. HT-29 cells were immunoblotted with antibodies targeting NCAPH2. Immunoblotting with antibodies against complex V and β-tubulin are shown to confirm the identity of mitochondrial and cytoplasmic fractions, respectively. NCAPH2 band intensities were normalized to the respective loading controls. NCAPH2 levels in isolated fractions from NCAPH2 shRNA-expressing cells were compared to levels in NT shRNA fractions, which were set to 100%. A representative of two independent experiments is shown. (B) Equal amounts of mitochondrial and cytoplasmic lysates were isolated from equal numbers of NT and NCAPG2 shRNA-expressing cells. HT-29 cells were immunoblotted with antibodies targeting NCAPG2. Immunoblotting with antibodies against complex V and β-tubulin are shown to confirm the identity of mitochondrial and cytoplasmic fractions, respectively. NCAPG2 band intensities were normalized to the respective loading controls. NCAPG2 levels in isolated fractions from NCAPG2 shRNA-expressing cells were compared to levels in NT shRNA fractions, which were set to 100%. A representative of two independent experiments is shown. (C) Equal amounts of mitochondrial and cytoplasmic lysates were isolated from equal numbers of NT and SMC2 shRNA-expressing HT-29 cells immunoblotted with antibodies targeting SMC2. Immunoblotting with antibodies against complex V and β-tubulin are shown to confirm the identity of mitochondrial and cytoplasmic fractions, respectively. SMC2 band intensities were normalized to the respective loading controls. SMC2 levels in isolated fractions from SMC2 shRNA-expressing cells were compared to levels in NT shRNA fractions, which were set to 100%. A representative of two independent experiments is shown.

To test whether other condensin II subunits localize to mitochondria, western blot analyses of mitochondrial lysates isolated from NT, NCAPH2, NCAPG2 and SMC2 shRNA-expressing cells were performed (Fig. 2A–C). These experiments confirmed that, like NCAPD3, NCAPH2 is detectable in mitochondrial lysates from HT-29 cells (Fig. 2A). Surprisingly, while results demonstrated NCAPG2 localization in the cytoplasm, NCAPG2 protein was not detected in mitochondrial lysates (Fig. 2B). In addition, we also detected SMC2 in mitochondrial lysates (Fig. 2C).

### NCAPH2 and SMC2 localize to the mitochondrial interior, whereas NCAPD3 localizes to the exterior

**Fig. 3.**
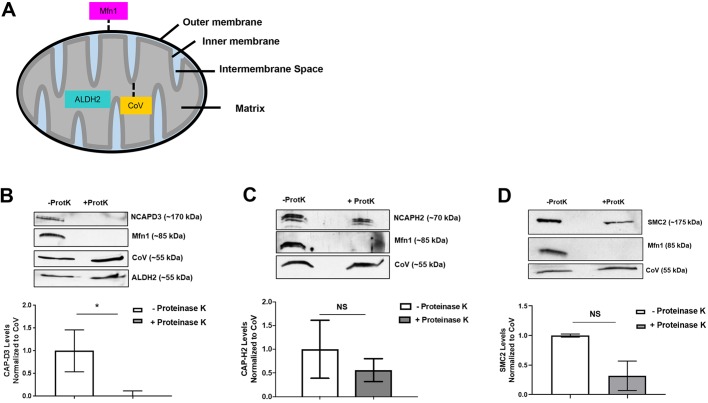
**NCAPD3 and NCAPH2 localize to different mitochondrial subcompartments.** (A) Diagram of mitochondria and localization patterns of mitochondrial proteins. (B) Equal amounts of mitochondrial lysate with or without proteinase K treatment were immunoblotted for NCAPD3. Mfn1 served as an outer membrane control, CoV served as an inner membrane control, and ALDH2 served as a matrix control. NCAPD3 levels were quantified in the lysates and normalized to the level of CoV. *n*=3 independent experimental replicates. (C) Equal amounts of mitochondrial lysate with or without proteinase K treatment were immunoblotted for NCAPH2. Mfn1 served as an outer membrane control, CoV served as an inner membrane control. NCAPH2 levels were quantified in the lysates and normalized to the level of CoV. *n*=3 independent experimental replicates. (D) Equal amounts of mitochondrial lysate with or without proteinase K treatment were immunoblotted for SMC2. Mfn1 served as an outer membrane control and CoV served as an inner membrane control. SMC2 levels were quantified in the lysates and normalized to CoV. *n*=2 independent experimental replicates. *P≤0.05; NS, not significant (Student's *t*-test).

Mitochondria are divided into four major sub-compartments: the outer membrane, inner membrane, intermembrane space and matrix (Fig. 3A). Proteinase K has been shown to cleave proteins present on the outer mitochondrial membrane. To determine which mitochondrial region NCAPD3, NCAPH2 and SMC2 proteins localize to, we first performed a proteinase K digestion on mitochondrial lysates isolated from HT-29 cells. As expected, NCAPD3, NCAPH2 and SMC2 were present in mitochondrial lysates prior to proteinase K digestion (Fig. 3B–D). However, following incubation with proteinase K, NCAPD3 protein was no longer detectable in mitochondrial lysates (Fig. 3B). This was also true for the control outer membrane protein mitofusin 1 (Mfn1) (Fig. 3A,B). The control inner membrane protein, CoV, and the control matrix protein, aldehyde dehydrogenase 2 (ALDH2) were protected from proteinase K digestion (Fig. 3B–D). Surprisingly, NCAPH2 and SMC2 both decreased following proteinase K digestion, but the decreases were not significant between experiments (Fig. 3C,D), suggesting that while NCAPD3 is present specifically on the exterior surface of mitochondria, NCAPH2 and SMC2 are capable of entering the organelle, and a small amount of NCAPH2 and SMC2 may also be present on the outer membrane. Taken together, these data suggest that condensin II subunits present in the mitochondrial interior are not part of the canonical condensin II holocomplex. Therefore, if NCAPH2 and SMC2 function to organize the mitochondrial genome in HT-29 cells, then they do so as part of a novel condensin II-like complex.

### Loss of condensin II CAP proteins leads to aberrant mitochondrial respiratory function and cellular ATP deficits

**Fig. 4.**
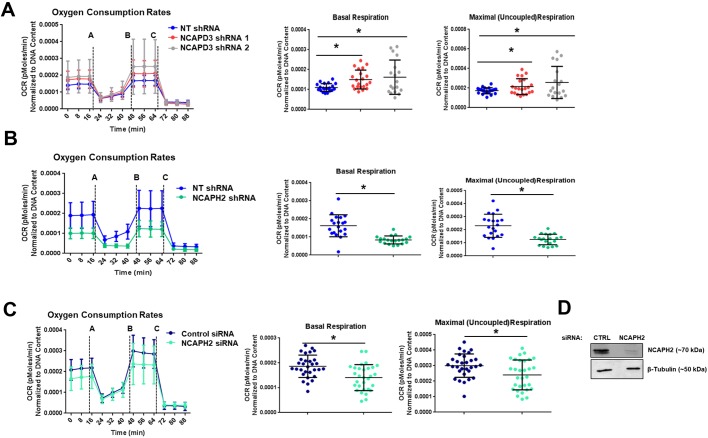
**Loss of condensin II CAP protein expression results in aberrant mitochondrial respiratory function.** Seahorse mitochondrial stress test assays were performed in (A) NT (*n*=21) and NCAPD3 (*n*=21, *n*=18) shRNA-expressing (three experimental replicates), (B) NT (*n*=20) and NCAPH2 (*n*=20) shRNA-expressing (two experimental replicates), and (C) control (*n*=30) and NCAPH2 (*n*=30) siRNA-transfected (three experimental replicates) cells. Cells were injected with drugs at the time points indicated: (A) 1 µM oligomycin; (B) 0.6 µM FCCP; (C) 1 µM antimycin A and rotenone. The first graph in each panel illustrates the full OCR profile, showing OCRs (*y*-axis) over time (*x*-axis). The second graph shows mitochondrial basal respiration rates. This value is calculated by subtracting non-mitochondrial respiration rates (OCR values post treatment with antimycin A and rotenone) from cellular respiration rates measured prior to addition of any mitochondrial inhibitory compounds. The final graph shows maximal (uncoupled) respiration rates. This value is measured after the addition of FCCP, a compound that uncouples oxygen consumption from ATP production. All values are normalized to DNA content by measuring the absorbance value at 485 nm relative to that at 535 nm using the Cyquant Cell Proliferation assay to account for possible differences in cell number. *P≤0.05. (Student's *t*-test). (D) Western blot analysis of NCAPH2 levels in control and NCAPH2 siRNA-transfected cells. β-tubulin served as a loading control.

Since NCAPH2 and NCAPD3 were found to be present at mitochondria, we wondered whether they might also influence mitochondrial function. Oxygen consumption rates (OCRs) were analyzed in HT-29 cells expressing NT, NCAPD3 (Schuster et al., 2015) or NCAPH2 (Ward et al., 2017) shRNA by performing a Seahorse mitochondrial stress test assay. Increases in both basal and maximal respiration rates were observed in NCAPD3-depleted cells (Fig. 4A). Surprisingly, decreased basal and maximal respiration rates were observed when NCAPH2 levels were depleted (Fig. 4B–D). Decreases in basal and maximal respiration rates were also observed in NCAPG2-depleted cells (Fig. S2).

**Fig. 5.**
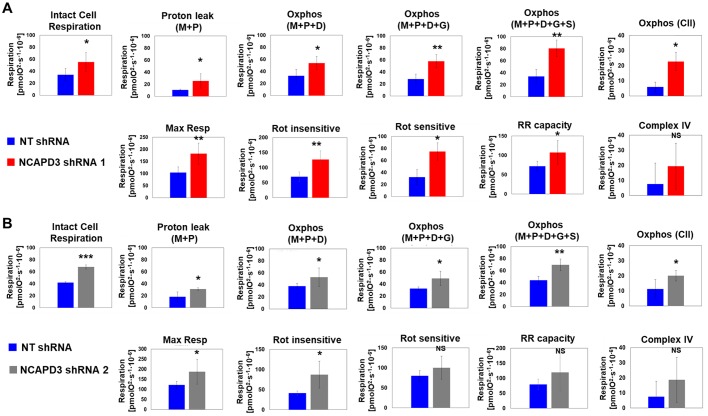
**NCAPD3-depleted cancer cells exhibit mitochondrial complex I-III dysfunction.** High-resolution respirometry was performed to measure OCRs in intact non-permeabilized HT-29 cells in mitochondrial respiration buffer, using the Oroboros instrument. Cells were permeabilized with digitonin and ETC complex-specific substrates and inhibitors were added, sequentially, as described in the Materials and Methods (Kumar et al., 2019; Pesta and Gnaiger, 2012). Proton leak and oxidative phosphorylation (OXPHOS) in response to complex I–III substrates malate (M), pyruvate (P), ADP (D), glutamate (G) and complex II substrate, succinate (S) were measured. complex II activity was determined by subtracting the OCRs following addition of malate, pyruvate and glutamate from the OCRs following addition of malate, pyruvate and glutamate and succinate. Maximum respiration (Max R) as well as reserve respiratory (RR) capacity (response to the protonophore FCCP) were quantified. Rotenone-sensitive and -insensitive respiration, followed by complex IV function were also measured. Experiments were performed in HT-29 cells induced to express (A) NCAPD3 shRNA 1 (red bars) or (B) NCAPD3 shRNA 2 (gray bars) and results were compared to HT-29 cells expressing NT shRNA (blue bars). Four biological replicates were performed for each cell line. *P<0.05, **P<0.005; NS, not significant (paired *t*-tests).

OCRs were also measured using the Oroboros respirofluorometer. Permeabilization of HT-29 cells expressing control NT shRNA resulted in decreased OCRs and the addition of substrates to test complex I function (pyruvate and malate, followed by ADP) showed that the oxidative phosphorylation pathway downstream of complex I was intact in these cells (Fig. 5A,B; Fig. S3). While the addition of glutamate did not significantly change the OCRs in these cells, the addition of succinate, a complex II substrate, resulted in a substantial increase in oxygen consumption, suggesting an intact oxidative phosphorylation pathway downstream of complex II. As expected, the addition of FCCP, an uncoupler of oxidation and phosphorylation, resulted in increased oxygen consumption that was inhibited by the complex I inhibitor, rotenone, and by the complex III inhibitor, antimycin A.

HT-29 cells expressing NCAPD3 shRNAs exhibited increased basal (‘Intact cell’) respiration rates, as previously demonstrated using the Seahorse protocols (Fig. 5A,B; Fig. S3). Like NT shRNA-expressing cells, following digitonin permeabilization, NCAPD3-deficient cells exhibited the expected reduction in OCRs, and, following the addition of complex I and II substrates, these cells exhibited increased OCRs (Fig. S3). However, OCRs consistently remained higher in NCAPD3-deficient cells as compared to controls (Fig. 5A,B). Interestingly, the addition of succinate, to measure complex II function, resulted in a significantly higher fold change in OCRs, as compared to control cells (Fig. S4), suggesting that complex II function may be especially sensitive to condensin II subunit depletion. In support of this idea, both complex II-dependent OCRs and rotenone-insensitive OCRs (a measure of complex I-independent respiration) were significantly increased in cells expressing either of the NCAPD3 shRNAs (Fig. 5A,B). In contrast, the addition of the complex III inhibitor antimycin A resulted in the expected near complete inhibition of oxygen consumption in both NT and NCAPD3 shRNA-expressing cells, with no significant differences in the NCAPD3-depleted cells. Likewise, complex IV-dependent OCRs were no different between NT and NCAPD3 shRNA-expressing cells (Fig. 5A,B).

To determine whether depletion of NCAPD3 also impacted mitochondrial function in primary cells, we inducibly expressed NT shRNA or NCAPD3 shRNA in ARPE-19 cells, which are primary retinal pigment epithelial cells, and again used the Oroboros instrument to measure OCRs (Fig. S5A). ARPE-19 cells were used owing to the fact that primary colon epithelial cells that can be easily genetically manipulated and then grown for several passages do not exist. We observed no significant differences in intact cell respiration in mitochondrial respiration buffer (Fig. S5B). In permeabilized cells, addition of the complex I substrates malate, pyruvate, ADP and glutamate also did not cause significant differences in oxygen consumption between control and NCAPD3-depleted cells. However, the addition of complex II substrate, succinate, again resulted in significant changes in OCRs in NCAPD3-deficient cells, although the changes were in the opposite direction as compared to HT-29 cells; NCAPD3-deficient ARPE-19 cells exhibited decreased oxygen consumption in response to succinate, while NCAPD3-deficient HT-29 cells exhibited increased oxygen consumption (Figs S4 and S5C). Finally, responses to the addition of the FCCP uncoupler or complex I inhibitor, rotenone, were not different between ARPE-19 cells expressing NT or NCAPD3 shRNA.

**Fig. 6.**
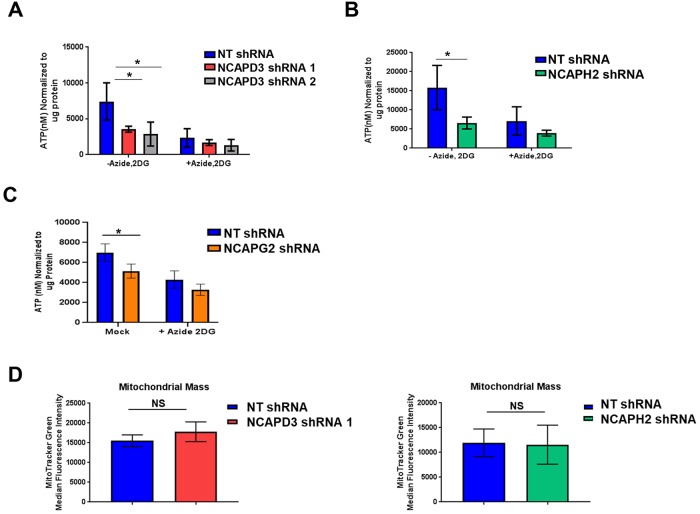
**Depletion of condensin II CAP proteins cause deficits in cellular ATP levels.** Cellular ATP levels were measured in (A) NT and NCAPD3 (B) NT and NCAPH2 and (C) NT and NCAPG2 shRNA-expressing cells after mock treatment (PBS) or treatment with inhibitors of ATP production (sodium azide, 10 mM and 2-deoxy-D-glucose, 6 mM). Levels were normalized to protein content. For all experiments, *n*=6 biological replicates. Each experiment was repeated twice. (D) Flow cytometry was performed in NT shRNA (blue bars), NCAPD3 shRNA 1 (red bars), and NCAPH2 shRNA (green bars)-expressing cells to measure median MitoTracker Green intensity, as a proxy for mitochondrial mass. For NCAPD3, *n*=6 biological replicates, two experimental replicates. For NCAPH2, *n*=4 biological replicates, two experimental replicates. *P≤0.05; NS, not significant (Student's *t*-test).

Taken together, these results suggest that condensin II CAP protein functions do influence mitochondrial respiration; however, this might involve different direct or indirect mechanisms, and these mechanisms may also be cell type-specific and/or differ between cancer cells and primary cells. Defects in oxygen consumption can be accompanied by changes in ATP levels. Interestingly, depletion of NCAPD3, NCAPH2 or NCAPG2 in HT-29 cells resulted in ATP deficits (Fig. 6A–C), suggesting that even though these subunits may affect mitochondrial function through different mechanisms, the end result for cellular metabolism could still be similar. To determine whether the defects in oxygen consumption or ATP levels in NCAPD3-deficient cells were due to changes in mitochondrial numbers, we stained cells with MitoTracker Green and performed flow cytometry to analyze mitochondrial mass. Results demonstrated no significant changes in mitochondrial mass between NT or NCAPD3 shRNA-expressing cells, suggesting that the observed changes in ATP levels and oxygen consumption are not caused by changes to mitochondrial mass (Fig. 6D).

HT-29 cells are a cancer cell line and possess inherent defects in metabolism, since cancer cells characteristically rely more heavily on glycolysis regardless of whether oxygen is present. This phenomenon is referred to as the Warburg effect (Vander Heiden et al., 2009; Boland et al., 2013). To test whether decreased condensin II levels affected glycolysis in this cell line, we performed extracellular acidification rate analyses (ECAR), again using the Seahorse Biosciences platform. Our data demonstrate no significant changes in glycolysis in NCAPD3- or NCAPH2-deficient cells, suggesting that changes in OCR occur independently of changes to glycolysis (Fig. S6).

### NCAPD3 and NCAPH2 differ in their ability to regulate mitochondria-associated gene transcription

**Fig. 7.**
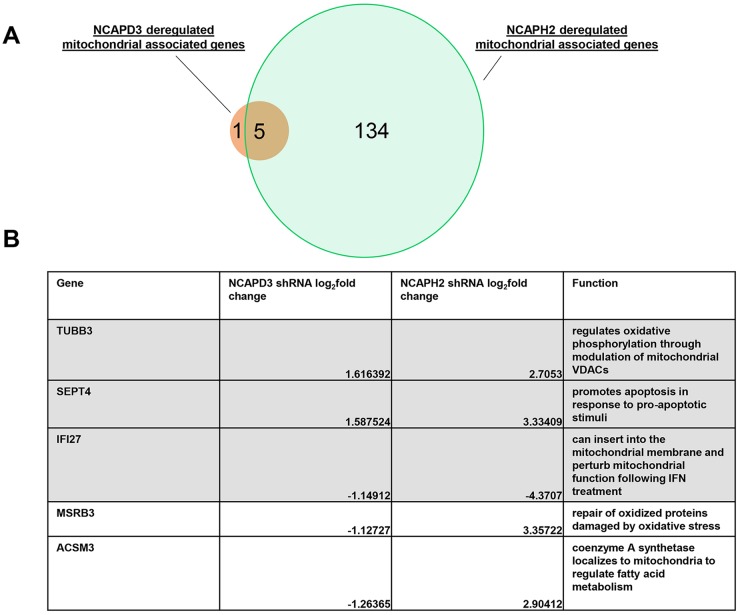
**NCAPD3 and NCAPH2 affect the expression of a few mitochondria-associated genes in a similar manner.** (A) Venn diagram comparing the shared deregulated mitochondrial associated genes in NCAPD3 (red circle) and NCAPH2 (green circle)-depleted cells. (B) Table detailing the shared deregulated mitochondrial associated genes, fold change and gene function.

Condensin II proteins have been demonstrated to regulate transcription, both directly and indirectly in several different organisms and several different cell types (Longworth et al., 2012; Deutschman et al., 2018; Schuster et al., 2013, 2015; Klebanow et al., 2016; Rawlings et al., 2011; Dowen et al., 2013; Yuen et al., 2017; Xu et al., 2006). To test whether NCAPD3 or NCAPH2 depletion could cause deregulation of mitochondrial and/or nuclear genes essential to mitochondrial function, RNA-seq experiments were performed in NT, NCAPD3 and NCAPH2 shRNA-expressing, subconfluent HT-29 cells. Unexpectedly, only 231 genes changed in NCAPD3-depleted cells, and only one of these genes, MT-TL1, a mitochondrial leucine tRNA, was encoded within the mitochondrial genome (Table S1). Since gene ontology (GO) analyses performed using the GO resource (http://geneontology.org/) bioinformatics platform did not identify any significantly changed gene programs or pathways (data not shown), we compared our list of differentially expressed nuclear-encoded genes to an inventory of mitochondrial-associated genes using the MitoCarta database (Pagliarini et al., 2008; Calvo et al., 2016) to determine whether any mitochondrial/metabolic pathways were affected as a result of NCAPD3 depletion. Surprisingly, only six (∼3%) of the 231 deregulated genes had been previously associated with mitochondria (Fig. 7A; Table S2).

The list of differentially expressed genes was much larger in NCAPH2-depleted HT-29 cells (Table S3). A total of 3177 genes were differentially expressed in these cells, and three mitochondria-encoded genes, MT-RNR1, MT-RNR2 and MT-TF, were present in this list. MT-RNR1 and RNR2 encode for the mitochondrial 12S and 16S rRNAs, respectively, and MT-TF encodes for the mitochondrial phenylalanine tRNA. GO analyses of deregulated genes in NCAPH2-deficient cells identified several potential cellular pathways that could be regulated by NCAPH2, but oxidative phosphorylation and mitochondrial function were not present within the list (Table S4). Comparison of this list of deregulated genes with the Mitocarta database revealed an overlap of 139 genes (Fig. 7A; Table S5). This list includes genes with roles in mitochondrial protein transport, mitochondrial signaling and ATP synthesis. These results suggest that, unlike NCAPD3, NCAPH2 may directly or indirectly play a significant role in transcriptional regulation of genes involved in various mitochondrial processes.

To determine whether NCAPD3 and NCAPH2 might regulate the same nuclear-encoded genes whose functions were important for mitochondrial biology, we compared the lists of mitochondria-associated differentially expressed genes in cells depleted for NCAPD3 or NCAPH2 (Fig. 7A,B). Three genes were found to be deregulated in a similar manner: TUBB3, IFI27 and SEPT4. TUBB3 encodes for β-tubulin III, a microtubule protein that has been shown to regulate oxidative phosphorylation through modulation of mitochondrial voltage-dependent anion channels (VDACs) (Puurand et al., 2019). IFI27 encodes for interferon-α inducible protein 27, which can insert into the mitochondrial membrane and contributes to the perturbation of normal mitochondrial function following interferon treatment (Rosebeck and Leaman, 2008). The SEPT4 gene is differentially spliced to produce a septin-like mitochondrial protein shown to promote apoptosis in response to pro-apoptotic stimuli (Gottfried et al., 2004). Two mitochondria-associated genes were deregulated in opposite directions in NCAPD3- and NCAPH2-deficient cells: ACSM3 and MSRB3. ACSM3 encodes for an acyl-coenzyme A synthetase, which localizes to mitochondria and also takes part in the first step of fatty acid metabolism (Ellis et al., 2015). MSRB3, which encodes for methionine sulfoxide reductase B3, localizes to mitochondria and is a member of a family of proteins involved in the repair of oxidized proteins damaged by oxidative stress (Cabreiro et al., 2008). These data suggest that NCAPD3 and NCAPH2 do not coordinately regulate a large transcriptional program to affect mitochondrial function.

### NCAPD3-depleted cells are more sensitive to mitochondrial stress

**Fig. 8.**
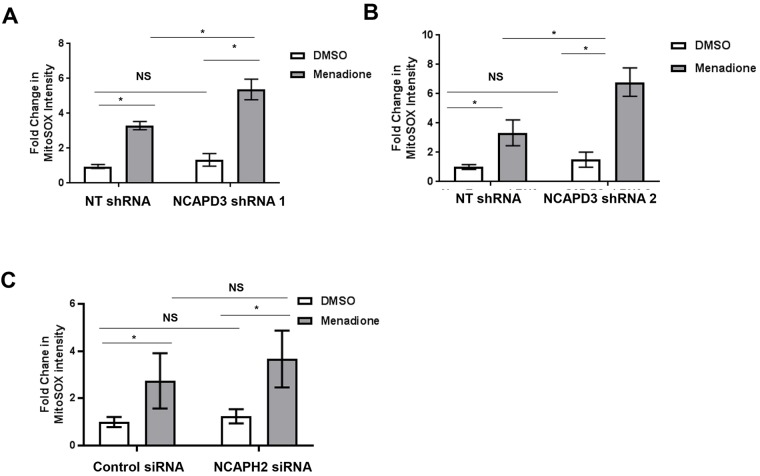
**NCAPD3 depletion results in sensitivity to mitochondrial oxidative stress.** (A,B) Flow cytometry analyses were performed to detect MitoSox Red in DMSO and menadione (50 μM)-treated NT and NCAPD3 shRNA-1-expressing cells. For NT and NCAPD3 shRNA-1-expressing DMSO-treated cells, *n*=4. For NT shRNA-expressing menadione-treated cells, *n*=4, and for NCAPD3 shRNA-1-expressing menadione-treated cells, *n*=3. For NT and NCAPD3 shRNA-2-expressing DMSO and menadione-treated cells, *n*=4. This experiment was repeated twice. (C) Flow cytometry analyses were performed to detect MitoSox Red in DMSO and menadione (50 μM)-treated NT and NCAPH2 siRNA-transfected cells. For control and NCAPH2 siRNA-transfected DMSO and menadione-treated cells, *n*=6. This experiment was repeated three times. *P≤0.05; NS, not significant (Student's *t*-test).

Mitochondrial respiratory dysfunction can result in increased generation of reactive oxygen species (ROS) (Murphy, 2009). If abundant ROS are not properly cleared, mitochondria can incur greater damage, exacerbating the dysfunctional phenotypes already observed, leading to oxidative stress (Murphy, 2009). Since we observed respiratory dysfunction following condensin II subunit depletion, we wondered whether loss of NCAPD3 or NCAPH2 would also result in increased ROS production. To test this, flow cytometry was performed to detect MitoSOX Red staining. MitoSOX Red is a mitochondria-targeted fluorogenic probe that produces red fluorescence when oxidized by superoxide within mitochondria, but not by other ROS or reactive nitrogen species (RNS). While we observed small increases in mitochondrial-specific ROS levels when NCAPD3 or NCAPH2 levels were depleted (Fig. 8A–C), these increases were not significant. We hypothesized that increasing oxidative stress in cells might exacerbate the underlying mitochondrial defects in NCAPD3- or NCAPH2-deficient cells, allowing us to uncover a role for these proteins in preventing ROS accumulation. Therefore, cells were treated with the redox cycling compound menadione to induce mitochondrial-specific ROS production. Excitingly, results showed significant increases in MitoSOX fluorescence intensity (i.e. ROS levels), in menadione-treated NCAPD3-depleted cells as compared to controls (Fig. 8A,B). Immunoblotting analyses of mitochondrial lysates from cells treated with menadione demonstrated that NCAPD3 levels did not increase at mitochondria (Fig. S7). NCAPD3 protein also remained on the outside of mitochondria following menadione treatment (data not shown). RNA-seq experiments performed in NT and NCAPD3 shRNA-expressing cells treated with menadione revealed only eight significantly deregulated genes (Table S6), suggesting that altered hypoxic gene transcription is not likely to be the reason for the impaired oxidative stress response in NCAPD3-depleted cells. In contrast to what was seen with NCAPD3 depletion, similar experiments performed in NCAPH2-depleted cells revealed no significant change in MitoSOX intensity in menadione-treated cells (Fig. 8C). Taken together, these data show that NCAPD3 promotes responses to oxidative stress in a manner that does not require massive changes to gene transcription and this function is not shared by the condensin II subunit NCAPH2.

## DISCUSSION

Condensin II is an essential complex whose multi-faceted functions in different subcellular compartments are just beginning to be explored. Our data demonstrate that the condensin II proteins NCAPD3, NCAPH2 and SMC2 localize to mitochondria. We also show that condensin II CAP subunits influence respiratory function and cellular ATP levels, and that NCAPD3 promotes efficient mitochondrial responses to oxidative stress. To date, most of the functions described for condensin II proteins have been attributed to the activity of the entire complex. However, the data presented here show that NCAPG2 is not present at mitochondria, suggesting that, if the localization of NCAPD3, NCAPH2 and SMC2 are important for their ability to regulate mitochondrial function, then these proteins are acting independently of the condensin II holocomplex.

Surprisingly, we were only able to detect NCAPD3 in mitochondria with an antibody that targeted internal residues of the protein, but not with an antibody recognizing C-terminal residues. This antibody also recognized a NCAPD3 doublet, suggesting that a modified version of the protein might exist in mitochondria. One possibility for why this antibody specifically recognizes mitochondrial NCAPD3 could be that the targeted residues are post-translationally modified in the cytoplasmic NCAPD3 pools, blocking the antibody from binding. Indeed, analysis of the NCAPD3 sequence using the ExPASy bioinformatics tool Prosite (Sigrist et al., 2012, 2002) identified putative phosphorylation and myristylation sites (Fig. S8). Additionally, the antibody targeting C-terminal residues (amino acids 1450–1498) of NCAPD3 specifically detected cytoplasmic pools of NCAPD3. This could mean that the mitochondrial form of NCAPD3 is C-terminally truncated and therefore cannot be recognized. This is supported by the fact that the mitochondria-specific form runs at a slightly lower molecular mass than the cytoplasmic form (data not shown). Future experiments designed to test these ideas will help to characterize the differences between mitochondrial and cytoplasmic NCAPD3 species.

While we observe condensin II protein localization to mitochondria and characterize the impacts that depletion of these proteins has on mitochondrial respiration and ROS clearance, we have not yet determined whether these functions are dependent on condensin II protein localization to mitochondria. In analyses with both MitoFates (Fukasawa et al., 2015) and Mitominer (Smith and Robinson, 2009, 2016; Smith et al., 2012), we were unable to identify any high confidence mitochondria-targeting sequences (MTSs) within the NCAPD3 or NCAPH2 protein sequence. Cell cycle-dependent post-translational modification of condensin II CAP subunits has been shown to regulate the association of condensin II proteins with DNA (Abe et al., 2011; Bakhrebah et al., 2015; Kagami et al., 2017), and it is therefore possible that cell cycle dynamics could also influence the localization of condensin II proteins to mitochondria. Another question arising from this work is whether NCAPD3 and NCAPH2 can transit back and forth between the nucleus and mitochondria. If this phenomenon does occur in cells, then these proteins could potentially facilitate signaling between the mitochondrial and nuclear stress response programs. Future studies aimed at identifying residues on NCAPD3 and NCAPH2 that are essential for mitochondrial targeting of these proteins would assist in answering these important questions.

Excitingly, our experiments using both the Seahorse and Oroboros instruments to measure oxygen consumption and test the function of ETC components revealed increased oxygen consumption and increased complex II function in NCAPD3-deficient HT-29 colon adenocarcinoma cells (Fig. 5; Figs S3 and S4). Interestingly, similar experiments performed in primary ARPE-19 cells demonstrated that NCAPD3 deficiency causes decreased oxygen consumption and decreased complex II function (Fig. S5). These results suggest that NCAPD3 may act as a checkpoint in cellular bioenergetic pathways; however, its potential roles in these pathways may be different between tumor-derived cells and primary cells, which have very different metabolic needs (Vander Heiden et al., 2009; Boland et al., 2013). Succinate is a product of substrate-level phosphorylation materialized in the citric acid cycle, and its metabolism heavily influences several other cellular metabolic pathways, as well as immune signaling and ROS homeostasis (West et al., 2011; Mills and O'Neill, 2014; Tretter et al., 2016). Future experimentation to determine whether the complex II dysfunction observed in NCAPD3-depleted cells results in changes to succinate levels or other intermediates in the tricarboxylic acid cycle will help elucidate the mechanisms by which condensin II subunit depletion impacts cellular metabolism and homeostasis.

Since condensin II regulates nuclear architecture (Hirano, 2012, 2016; Kalitsis et al., 2017; Ono et al., 2004; Li et al., 2015a; Rowley et al., 2019; Bauer et al., 2012; Hartl et al., 2008; Joyce et al., 2012; Rosin et al., 2018) and gene expression in response to stress (Li et al., 2015a; Longworth et al., 2012; Schuster et al., 2015), it is reasonable to think that changes in nuclear gene expression might be responsible for the defects in mitochondrial function that occur following condensin II protein depletion. However, our data show that very few genes associated with mitochondrial function change as a result of NCAPD3 knockdown. Interestingly, our studies did identify *PDK4* as a downregulated gene in NCAPD3-deficient cells. PDK4 facilitates the phosphorylation and subsequent inactivation of the pyruvate dehydrogenase complex (PDC), inhibiting the conversion of pyruvate into acetyl-CoA. This prevents the entry of acetyl-CoA into the TCA cycle, decreasing the generation of NADH and leading to lower respiration rates (Zhang et al., 2014). Therefore, it is possible that downregulation of PDK4 in NCAPD3-deficient cells could lead to elevated basal and maximal respiration (Fig. 5).

Strikingly, NCAPH2 depletion in HT-29 cells resulted in significant changes in gene expression to over 3000 genes, which is more than 10-fold greater than the number found for NCAPD3-depleted cells. Several possibilities could explain these results. First, it is possible that NCAPH2 and NCAPD3 possess functions outside of the canonical condensin II complex that contribute to the deregulation of gene transcription, indirectly. Another possibility is that multiple forms of condensin II complexes exist, and some of these forms are mutually exclusive for NCAPD3 and NCAPH2. This idea is supported by evidence in *Drosophila* showing that endogenous dCAP-D3 and dCAP-H2 do not co-precipitate in ovaries (Herzog et al., 2013). A third possibility is that NCAPH2 may act redundantly with another protein to regulate gene expression in NCAPD3-depleted cells. This cannot be true for all differentially expressed genes in NCAPD3-deficient cells, however, since more than 50% of these genes are unique to NCAPD3-depleted cells and are not deregulated in NCAPH2-depleted cells (Tables S1 and S3). Finally, it is also possible that NCAPD3 and NCAPH2 depletion affect the cell cycle differently in these cells, and/or cause genomic instability in different genomic regions, thus affecting the expression of genes on different chromosomes.

NCAPH2 depletion resulted in the deregulation of more mitochondria-associated genes than NCAPD3 depletion, and several of these genes have been previously reported as being essential for mitochondrial function. These data suggest that NCAPH2 may be regulating mitochondrial function through direct or indirect control of gene expression. The fact that only five mitochondria-associated genes are deregulated in both NCAPH2- and NCAPD3-depleted cells, with two of those genes being deregulated in opposite directions (Fig. 7B) again supports the idea that NCAPH2 and NCAPD3 are most likely working through separate mechanisms to regulate mitochondrial function. However, experiments testing whether mitochondrial function is rescued after correcting the expression levels (through knockdown or overexpression) of these five genes in NCAPH2- and NCAPD3-deficient cells will determine whether any of these genes are rate limiting for the observed mitochondrial phenotypes. Our RNA-seq data also revealed that three mitochondrial genes, MT-RNR1, MT-RNR2 and MT-TF, were all upregulated in NCAPH2-deficient cells. Interestingly, all of these genes are located sequentially within the mitochondrial genome; however, MT-RNR1 and MT-RNR2 are transcribed from the HSP1 promoter while MT-TF is the first gene of the heavy-strand polycistron transcribed from the HSP2 promoter (Mposhi et al., 2017). While we have shown that NCAPH2 is present in the interior of mitochondria, whether condensin II proteins participate in organizing the mitochondrial genome and regulating mitochondrial-encoded gene expression directly remains an open question.

Recent studies in *Drosophila* larval wing discs have shown that depletion of mitotic arrest deficient 2 (Mad2) induces chromosome instability which makes cells more sensitive to metabolic stress (Shaukat et al., 2015). Since CAP-D3 protein depletion results in aneuploidy and chromosome instability in several organisms (Woodward et al., 2016), it is possible that NCAPD3 depletion affects cellular responses to oxidative stress by disrupting genome integrity. If NCAPD3-deficient cells were already experiencing high levels of stress due to genome instability, then the addition of menadione might cause them to be incapable of efficiently responding to or clearing ROS. Experiments designed to elucidate the mechanisms and pathways by which elevated mitochondrial ROS occurs in NCAPD3-depleted cells will shed light on these interesting questions.

In previous immunoprecipitation mass spectrometry experiments, our laboratory identified predicted mitochondrial proteins as NCAPD3-binding partners. Interestingly, many endoplasmic reticulum (ER)-associated proteins were also identified (Ward et al., 2017). Mitochondria and ER are tightly linked and come into contact with one another at junctions termed ER–mitochondria contact sites (Vance, 1990; Rizzuto et al., 1998; Copeland and Dalton, 1959; Tubbs and Rieusset, 2017). These sites have been implicated in a variety of processes including Ca^2+^ signaling (Rizzuto et al., 2012; Tubbs and Rieusset, 2017; Lee et al., 2018; Tadic et al., 2014; Krols et al., 2016), innate immune signaling (Tubbs and Rieusset, 2017; Weinberg et al., 2015; Martinvalet, 2018; Horner et al., 2011; Bantug et al., 2018) and mitochondrial division (Lewis et al., 2016; Murley et al., 2013; Friedman et al., 2011), all of which can impact respiratory function. While NCAPD3 has a known role in the regulation of innate immune responses to bacterial infection (Longworth et al., 2012; Schuster et al., 2015), it has yet to be linked to either Ca^2+^ signaling or mitochondrial division.

Finally, condensin II malfunction has been implicated in a myriad of diseases including cancer (Deutschman et al., 2018; Leiserson et al., 2015; Zhan et al., 2017; Coschi et al., 2014; Ham et al., 2007), microcephaly (Martin et al., 2016) and inflammatory bowel disease (Schuster et al., 2015). All of these diseases have been linked to mitochondrial dysfunction (Condie et al., 2010; Suzuki and Rapin, 1969; Wallace, 2012; Novak and Mollen, 2015). Interestingly, our RNA-seq data revealed that NCAPD3 depletion results in the downregulation of the MT-TL1 gene, which is encoded in the mitochondrial genome. MT-TL1 is one of the most common pathogenic mitochondrial gene mutations, and is a genetic cause of mitochondrial encephalomyopathy with lactic acidosis and stroke-like episodes (MELAS) (Goto et al., 1990; Chinnery et al., 2000; Majamaa et al., 1998; Uusimaa et al., 2007). Patients with mutated MT-TL1 often show severe respiratory chain defects (Fornuskova et al., 2008; Dubeau et al., 2000). While the exact mechanisms of how MT-TL1 mutation causes disease have not been determined, studies show that cells exhibit lower levels of aminoacylation of mt-tRNA^Leu(UUR)^, deficiency in mitochondrial protein translation and lower steady state levels of respiratory chain complexes (Fornuskova et al., 2008; Sasarman et al., 2008). It will be interesting to determine whether MT-TL1 is rate limiting for the mitochondrial phenotypes observed in NCAPD3-depleted cells, and whether links exist between MELAS patients and NCAPD3 levels. Furthermore, future investigations into the mechanism by which condensin II proteins regulate mitochondrial function and responses to oxidative stress will help to determine whether the ability of these proteins to regulate cellular metabolism is vital to the prevention of disease.

## MATERIALS AND METHODS

### Fly stocks

Stocks were maintained at 25°C on standard dextrose medium. *w*; P{sqh-EYFP-Mito}3 (LaJeunesse et al., 2004; Bloomington Stock Center 7194) larvae expressing YFP at the mitochondrial membrane were used to determine condensin II subunit subcellular localization patterns.

### Cell lines

HT-29 cells were cultured in RPMI-1640 medium with 10% fetal bovine serum (FBS) and 1% penicillin-streptomycin. NT and NCAPD3 shRNA-expressing lines were previously made and validated (Schuster et al., 2015). NCAPH2 shRNA-expressing lines were previously made and validated (Ward et al., 2017). NCAPG2 shRNA-expressing HT-29 cells and NT shRNA and NCAPD3 shRNA-expressing ARPE-19 cells were made using lentiviral transduction using custom viral particles produced with the pLKO-puro-IPTG-3xLacO vector (Sigma-Aldrich) using the same method as reported in Ward et al. (2017). In the absence of isopropyl β-D-1-thiogalactopyranoside (IPTG), an analog of lactose (LacI) binds to LacO and prevents the expression of the shRNA. The presence of IPTG drives a conformational change of the allosteric LacI repressor, such that it is released from the LacO modified human U6 promoter, resulting in shRNA expression. All parental cell lines were obtained from the ATCC and are routinely tested on a monthly basis for mycoplasma contamination.

### IPTG treatment

Cells were seeded and allowed to attach overnight. Cells were treated for 48 h in standard growth medium with 1 µM (NCAPD3 and NCAPH2 shRNA-expressing cells) or 2 µM (NCAPG2 shRNA-expressing cells) IPTG. To induce SMC2 knockdown, cells were treated with 2 µM IPTG for 24 h.

### siRNA transfection

siRNA-mediated knockdown was performed as previously described in Ward et al. (2017). Briefly, Lipofectamine 2000 was used according to manufacturer's instructions to transfect control (40 nM; D-0012606-13-05, siGENOME NT siRNA pool) and NCAPH2 (40 nM; D-016186-03-005, siGENOME NCAPH2) siRNA.

### RNA isolation

Total RNA was isolated from human cells using the Qiagen Mini RNeasy Kit according to the manufacturer's protocol.

### Immunofluorescence

In *Drosophila*, all tissues were fixed at room temperature in 4% paraformaldehyde in phosphate-buffered saline (PBS) with 0.5% Triton X-100 (0.05% PBT) for 25 min. Tissues were blocked in 0.1% PBT with 1% BSA and incubated overnight with primary antibody in blocking buffer with rocking at 4°C. The antibody against dCAP-D3 was previously purified and validated in Longworth et al. (2008) and used at a dilution of 1:50. Secondary antibodies (Alexa Fluor conjugated; Thermo Fisher Scientific) were used at a 1:500 dilution. Tissues were mounted in Vectashield with DAPI. Human cells were fixed with 4% formaldehyde. Cells were blocked in PBS with 0.5% NP-40 1% BSA and incubated overnight in a humid chamber with primary antibody in blocking buffer. The antibody against NCAPD3 (Bioss; bs-7734R, AG10096644) was used at 1:500, and against CoV (Thermo Fisher Scientific; 439800, L5135) at 1:500. Secondary antibodies (Alexa Fluor conjugated; Thermo Fisher Scientific) were used at a 1:500 dilution. Cells were mounted in Vectashield with DAPI. All imaging was performed using a Leica SP5 confocal/multi-photon microscope.

### OCR assays using the Seahorse mitochondria stress test assay

200,000 cells were plated in a six-well tissue culture plate and allowed to attach overnight. To induce knockdown, cells were either treated with IPTG or transfected with siRNA. After 24 h, cells were collected and counted, and 50,000 were reseeded onto a Seahorse XF24/XFe24 assay plate (in IPTG containing medium if necessary). After 24 h cells were washed with assay medium (Seahorse XF RPMI supplemented with 1 mM glutamine, 25 mM glucose and 1 mM sodium pyruvate; all upstream templates of oxidative phosphorylation). Following washes, cells were incubated in 560 µl of assay medium at 37° in the absence of CO_2_ for 1 h, to equilibrate cells to the atmosphere of the Seahorse Bioanalyzer. After incubation, cells were stressed with different mitochondrial inhibitors, and OCRs were measured using the Seahorse XF24/XFe24 analyzer (Agilent). Drug injections are provided in the order in which they occurred along with a brief description of their purpose as provided by the manufacturer's protocol below. From Port A, the ATPase inhibitor, oligomycin was injected to a final concentration of 1 µM. Addition of oligomycin results in a decrease in OCRs. Next, from Port B, Carbonyl cyanide-p-trifluoromethoxyphenylhydrazone (FCCP) was injected to a final concentration of 0.6 µM. FCCP is a proton uncoupler that dissipates the mitochondrial protein gradient and dissociates ATP production from respiration, resulting in an increase in respiration rates to a maximal level. Finally, from Port C, antimycin A and rotenone were injected into to a final concentration of 1 µM. Antimycin A and rotenone block complex III and complex I, respectively. In inhibiting ETC activity, antimycin A and rotenone addition results in decreased OCR, such that any read values at this point are a measure of non-mitochondrial respiration. OCR was normalized to cell number through the CyQuant Proliferation assay (Thermo Fisher Scientific, C7026).

### OCR measurements using the Oroboros instrument

To complement the basal and maximum respiration quantified by the Seahorse protocol, we evaluated mitochondrial responses in our cellular models to a standard substrate uncoupler inhibitor titration (SUIT) protocol, using a sensitivity respirofluorometer, as previously described (Kumar et al., 2019; Pesta and Gnaiger, 2012). In brief, HT-29 cells expressing NT shRNA, NCAPD3 shRNA or NCAPH2 shRNA were permeabilized by digitonin and maintained in mitochondrial respiration medium MiR05. The mitochondrial substrates malate, pyruvate and glutamate, which generate the complex I substrate, NADH, were added. This was followed by the addition of complex II substrate, succinate. Proton leak respiration was quantified in the presence of pyruvate and malate, without ADP. Oxidative phosphorylation capacity and ATP synthesis-linked OCRs were quantified following addition of ADP and the substrates mentioned above. Phosphorylation restriction of the ETC was quantified by uncoupling oxidation and phosphorylation with FCCP, a protonophore, to determine the maximum oxidation capacity or theoretical maximum respiration. The rotenone-sensitive rate of oxygen consumption was used to measure the uncoupled complex I rate of oxygen consumption; this was determined by calculating the absolute reduction from the maximum uncoupling rate of both complex I and II substrates. The rotenone-insensitive rate of oxygen consumption was measured to determine the rate of oxygen consumption that was independent of uncoupled complex I. The antimycin A-insensitive rate is a measure of non-mitochondrial residual OCR. The uncoupled complex IV oxidation rate is a measure of sodium azide insensitive oxygen consumption, and was calculated by subtracting the OCR following azide addtion from the OCR in response to tetramethyl phenylene diamine (TMPD) plus ascorbate. Exogenous cytochrome was not added in the protocols to test complex IV function, since this would have masked any endogenous defects present in condensin II subunit-deficient cells. Throughout the protocols, OCRs were calculated by recording oxygen concentration and flow rates at 2 s intervals, using the DatLab2 software (Oroboros, Innsbruck, Austria), as previously described (Kumar et al., 2019; Pesta and Gnaiger, 2012). To compare mitochondrial respiration in response to different substrates and inhibitors, oxygen consumption was corrected for residual oxygen consumption. All experiments included at least four biological replicates after calibration of the oxygen sensors and instrument background corrections. Data is expressed as mean±s.d., and *P*-values were calculated using paired *t*-tests.

### ECAR assays

200,000 cells were handled and treated as described above for OCR assays. On the day of the assay, cells were washed with Seahorse XF RPMI supplemented with 1 mM glutamine. Glucose was left out of the medium so that basal rates of glycolysis could be measured upon stimulation with glucose at the start of the assay. Sodium pyruvate, a product of glycolysis that feeds into the TCA cycle and oxidative phosphorylation, was also not supplemented in the medium. ECARs were measured using a Seahorse XF24/XFe24 analyzer. Drug injections are provided in the order in which they occurred along with a brief description of their purpose as provided by the manufacturer's protocol below. First from Port A, glucose was injected to a final concentration of 10 mM to stimulate glycolysis. Next, from Port B, oligomycin was injected to a final concentration of 1 µM. As addition of oligomycin blocks ATP synthase, ATP production can only occur through glycolysis, resulting in increased ECAR values. Finally, from Port C, 2-deoxy-D-glucose is injected to a final concentration of 50 mM. 2-Deoxy-D-glucose is an analog of glucose that inhibits glycolysis, which results in decreased ECAR values. ECAR was normalized to cell number using the CyQuant Proliferation assay (Thermo Fisher Scientific, C7026).

### CyQuant proliferation assays

After the completion of OCR or ECAR assays, excess medium was aspirated off and the plate was turned upside down to empty out/blot off all remaining liquid. Plates were then placed at −80°C for at least 2 h. Following freezing, plates were removed from the freezer and allowed to thaw at room temperature. CyQuant GR dye was used at a final concentration of 2×; 200 μl of sample buffer was added to each well and the plate was covered and incubated for 5 min at room temperature. Post incubation, samples were mixed and transferred to wells on a clear 96-well flat-bottomed plate. Absorbance was read using a Perkin Elmer Victor2 1420 multi-labeled plate counter.

### ATP detection

Cells were washed and then incubated in Seahorse XF RPMI medium with 1 mM glutamine, 25 mM glucose and 1 mM sodium pyruvate. Cells were then mock treated with PBS or with 2-deoxy-D-glucose and sodium azide at final concentrations of 6 mM and 10 mM for 1 h to block ATP production via glycolysis and oxidative phosphorylation, respectively. Cells were then washed with 1× PBS, harvested in 1× reporter lysis buffer (RLB, Promega), flash frozen in liquid nitrogen, boiled at 99°C for 15 min, centrifuged at 16168 ***g***, and supernatant retained. 25 µl of supernatant was used to quantify protein concentration using a Pierce BCA assay (Thermo Fisher 23227) for normalization. Another 25 µl was taken and further diluted in 500 µl of 1× RLB. 10 μl of this dilution was used to detect cellular ATP levels in 90 µl of reaction buffer using a luciferase-based ATP determination kit (Thermo Fisher Scientific, A22066). Luminescence was read using a Perkin Elmer Victor2 1420 multi-labeled plate counter. All reactions were performed in triplicate and a standard curve was generated using known amounts of ATP to calculate ATP concentrations in the samples.

### Isolation of mitochondria

Cells were counted, and mitochondria isolated from an equal number of cells using the Mitochondrial Isolation Kit for Cultured Cells (Thermo Fisher Scientific, 89874) following the manufacturer's instructions. Centrifugation was performed at 3000 ***g*** for 15 min to obtain a more purified mitochondrial fraction. Isolated mitochondria were lysed in equal volumes of 2% CHAPS in TBS. The cytoplasmic fraction was also retained.

### Proteinase K digestion

Mitochondria were isolated as described above. Pelleted mitochondria were resuspended in a buffer consisting of 250 mM sucrose, 20 mM HEPES-NaOH (pH 7.9), 10 mM KCl, 1.5 mM MgCl_2_, 1 mM EDTA, 1 mM EGTA and 1:300 of a volume of proteinase inhibitor (Sigma 8340) and incubated at room temperature for 30 min in the presence of proteinase K at a final concentration of 50 µg/ml. PMSF was added at a final concentration of 5 mM to terminate the reaction. Triton X-100 was then added to the reaction at a final concentration of 1% to lyse mitochondria. Samples were centrifuged and supernatant was retained.

### Immunoblotting

Protein content was measured using a standard Pierce Bradford assay. Equal amounts of lysate were run on an SDS-PAGE gel. Blots were incubated with the antibodies against the following proteins at the noted dilutions in 0.1% PBT with 5% BSA: NCAPD3 (Bethyl; A-300-604A), 1:1000; NCAPD3 (Bioss; bs-7734R, AG10096644), 1:1000; NCAPH2, (Bethyl; A302-275A), 1:1000–1:10,000, NCAPG2 (Abcam; ab70350, GR300635-21), 1:2000; SMC2 (Abcam, ab10412), 1:5000, complex V (Thermo Fisher Scientific, 439800/L5135) 1:50,000, tubulin (Cell Signaling, 2146s, Lot7) 1:1000–1:10,000, Mfn1 (Abcam, ab57602/GR3239895-1), 1:250; ALDH2 (Abcam, ab70917/GR305332-3), 1:500. Secondary antibodies were used at 1:5000.

### Oxidative stress assays

Cells were incubated with DMSO or 50 µM menadione (Sigma) in standard growth medium for 30 min. For western blot analyses, mitochondria were isolated from DMSO- and menadione-treated cells. For RNA-seq, RNA was isolated and experiments performed as described below. For flow cytometry analyses, cells were incubated in fresh medium with either DMSO or 2.5 μM MitoSOX for 30 min. After incubation, cells were washed with PBS and detached from the plate with cell dissociation buffer (Thermo Fisher Scientific, 131510140). Cells were collected in PBS with 0.2% BSA and passed once through a cell strainer. Flow cytometry analysis was performed using an LSRFortessa with excitation at 510 nm and emission at 580 nm. 20,000 events were recorded.

### MitoTracker

Cells were washed once with PBS and then incubated with 200 nM MitoTracker Green FM (Thermo Fisher Scientific, M7514) in RPMI medium without FBS for 30 min and flow cytometry analysis performed. After incubation, cells were washed with PBS and detached from the plate with cell dissociation buffer (Thermo Fisher Scientific, 131510140). Cells were collected in PBS with 0.2% BSA and passed once through a cell strainer. Flow cytometry analysis was performed using an LSRFortessa with excitation at 490 nm and emission at 516 nm. 20,000 events were recorded.

### RNA-seq

All experiments were performed in biological triplicate. RNA was isolated as described following 30 min of DMSO or menadione (50 µM). cDNA libraries (50 base pair, single end) were made at the University of Chicago Genomics Core and sequenced on an Illumina HiSeq2500, according to standard protocols. Raw data files are available under the NCBI Gene Expression Omnibus accession number GSE139540. Reads were aligned to the human genome (GRCh38/hg38) using STAR (v.2.5.3a; Dobin et al., 2013) in gene counting mode (--quant ModeGeneCounts) with Gencode (v.27) annotations. After first-pass alignment, splice junctions were combined and filtered to remove non-canonical junctions and junctions covered by <10 reads prior to second-pass alignment. Second-pass counts were loaded into R (v.3.3.3, The R Project for Statistical Computing, https://www.r-project.org/) and analyzed with DESeq2 (v.1.14.1; Love et al., 2014). To offset uneven sample variance, fdrtool (Strimmer, 2008) was used to re-estimate false discovery rates where *P*-values were not uniformly distributed outside of the low *P*-value peak that includes differentially expressed genes. Genes with adjusted *P*<0.05 and fold change >2 were deemed to be differentially expressed. GO analysis was performed using the Gene Ontology Resource (http://geneontology.org/) bioinformatics platforms using Bonferroni correction for multiple testing.

### Statistics

Unless otherwise noted, all averages are reported as mean±s.d. *P*-values (unless otherwise stated) are two-tailed and were calculated using an unpaired Student's *t*-test.

## Supplementary Material

Supplementary information
