# Supplementary material for: Condensin II protein dysfunction impacts mitochondrial respiration and mitochondrial oxidative stress responses
Source: J Cell Sci. 2019 Nov 20;132(22):jcs233783. doi: 10.1242/jcs.233783 (PMC6899004; doi:10.1242/jcs.233783)
Supplement: Supplementary information [file joces-132-233783-s1.pdf]

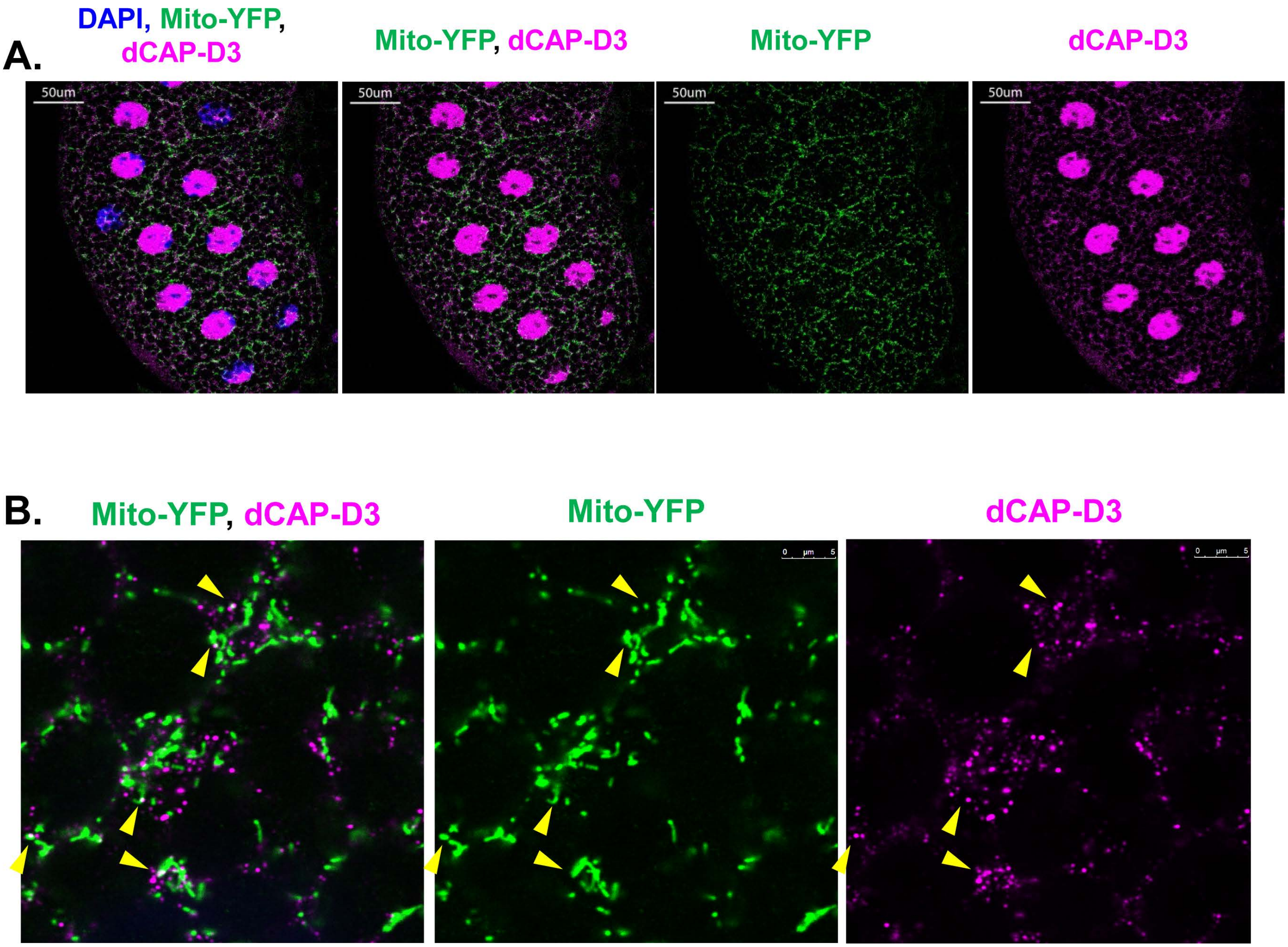

Figure S1: *Drosophila* CAP-D3 localizes to mitochondria *in vivo*.

Immunofluorescence analyses performed in w; P{sqh-EYFP-Mito}3 third instar larval salivary glands expressing sqh-EYFP-Mito (green) to label mitochondria. Images are of a single plane. A) Image of the distal portion of the salivary gland showing nuclear dCAP-D3 (magenta) localization. Nuclei are DAPI stained (blue). B) Magnified image of the same salivary gland, focused on the cytosolic region between individual salivary gland cells. Yellow arrows point out colocalization of dCAP-D3 with sqh-EYFP-Mito (mitochondria).

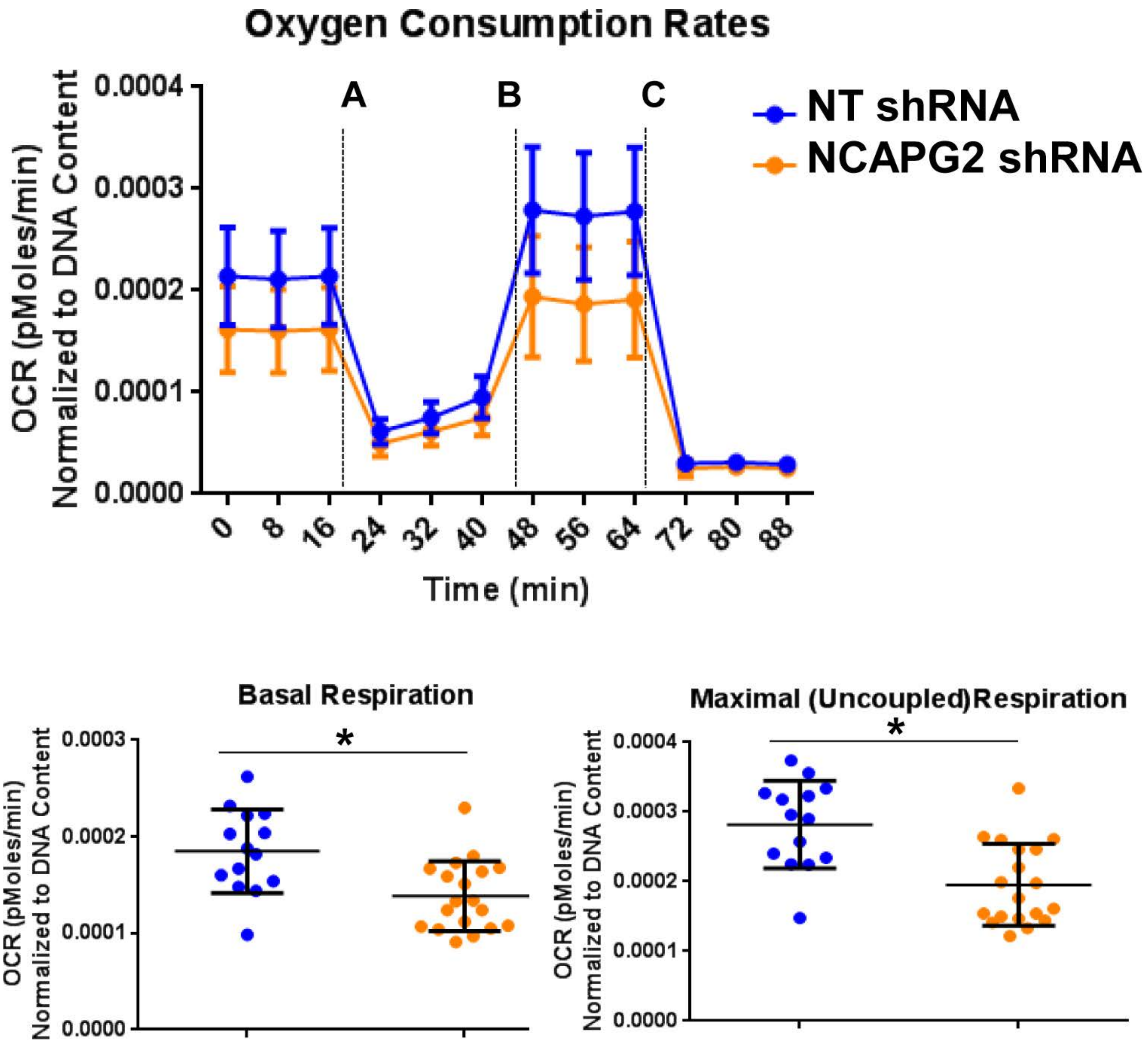

Figure S2. Loss of NCAPG2 results in aberrant mitochondrial respiratory function

Seahorse Mito Stress Test assays were performed in Non- Non-Target (n=14) and NCAPG2 (n=19) shRNA expressing cells. Cells were injected with drugs at the time points indicated A- 1μM Oligomycin, B- 0.6μM FCCP, C- 1μM Antimycin A/Rotenone. Graphs illustrate the full oxygen consumption rate profile, basal respiration rates, and maximal (uncoupled) respiration rates. The first graph in each panel illustrates the full

Journal of Cell Science • Supplementary information

oxygen consumption rate profile, showing oxygen consumption rates (y-axis) over time (x-axis). The second graph shows mitochondrial basal respiration rates. This value is calculated by subtracting non-mitochondrial respiration rates (OCR values post-treatment with antimycin A/rotenone) from cellular respiration rates measured prior to addition of any mitochondrial inhibitory compounds. The final graph shows maximal (uncoupled) respiration rates. This value is measured after the addition of FCCP, a compound that uncouples oxygen consumption from ATP production. All values are normalized to DNA content by measuring the A485/A535 absorbance ratio using the Cyquant Cell Proliferation assay. The experiment was repeated three times and a student t-test was used to determine significance, $p \leq 0.05$.

Journal of Cell Science • Supplementary information

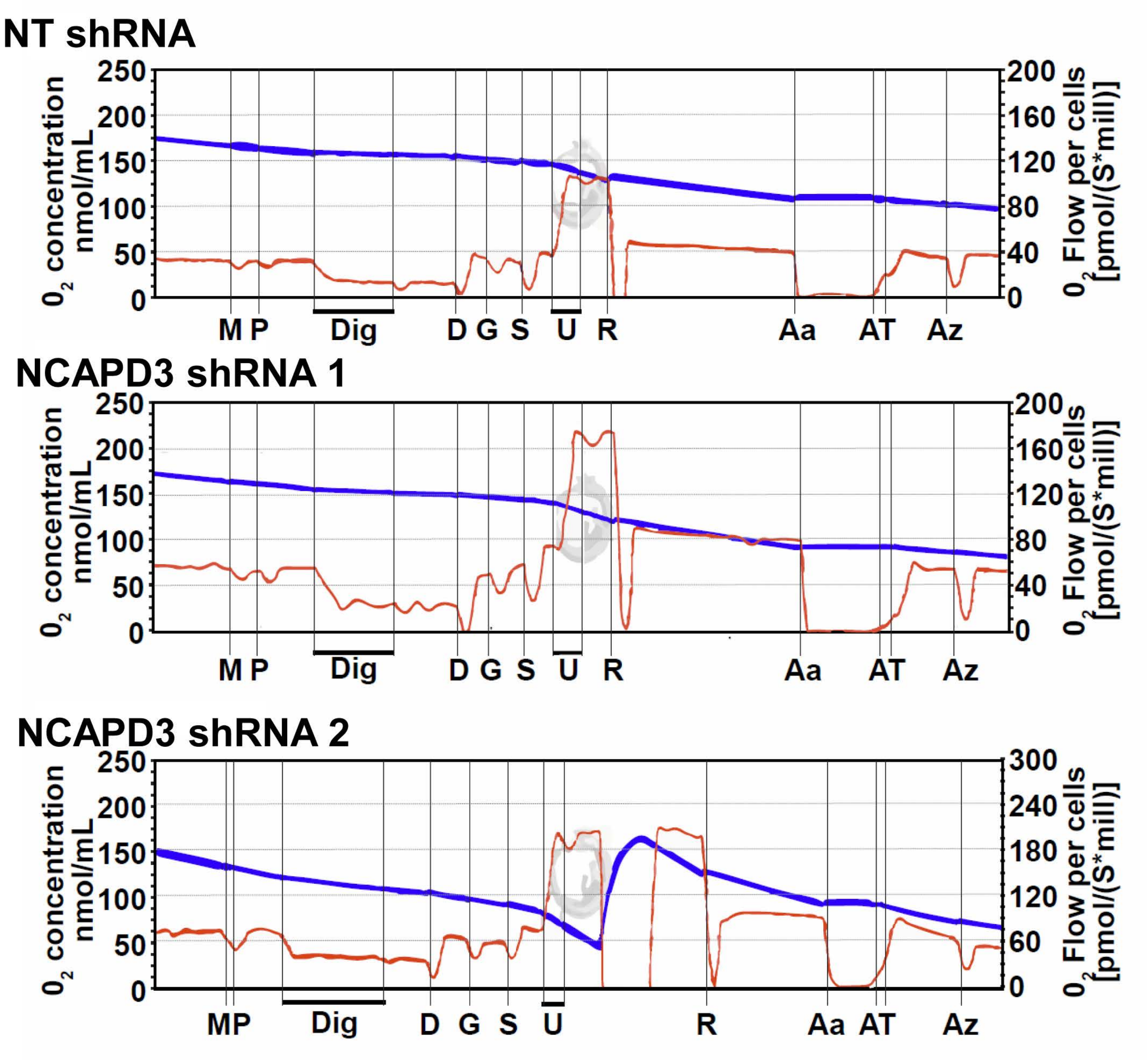

Figure S3. Representative tracings of high-resolution respirometry to quantify respiration of permeabilized HT-29 cells. Oxygen concentrations (blue lines) and oxygen consumption rates (red lines) were measured using the Oroboros instrument. After initial stabilization, 2mM malate (M) and 2.5 mM pyruvate (P) were added. This was followed by 4.1 μM digitonin (Dig) to permeabilize the cell membrane and permit entry of mitochondrial substrates inside the cells, without losing the integrity of cells or

Journal of Cell Science • Supplementary information

mitochondria, 2.5 mM ADP (D), 10 mM glutamate (G), 10 mM succinate (S), 2 μM increments of FCCP (U) for measuring maximum respiration. This was followed by addition of 375 nM rotenone (R), 125 nM antimycin A (Aa), 2 mM ascorbate and 2 mM TMPD (tetramethyl p-phenylene diamine) (AT) to test complex IV activity. Finally, 50 mM sodium azide (Az) was added to inhibit complex IV activity. Experiments were performed in HT-29 cells induced to express Non-target (NT) shRNA, or one of two different shRNAs targeting NCAPD3.

Journal of Cell Science • Supplementary information

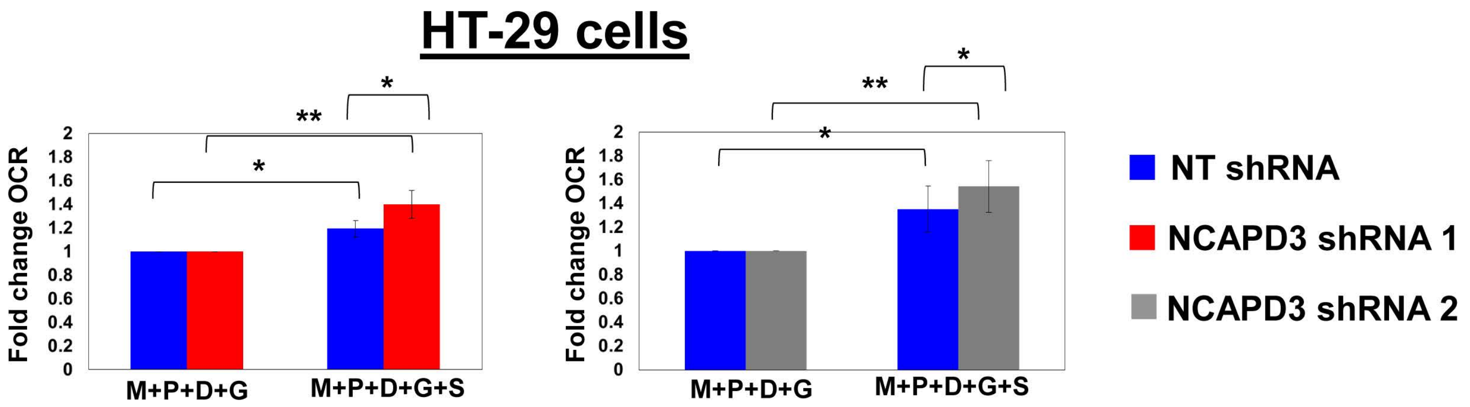

Figure S4. NCAPD3 deficiency results in Complex II dysfunction in tumor-derived cells. Fold change in oxygen consumption rates (OCR) were measured in HT-29 cells induced to express Non-target (NT)(blue bars), NCAPD3 shRNA 1 (red bars) or NCAPD3 shRNA 2 (gray bars) by normalizing the OCR following addition of succinate to the OCR following addition of glutamate (which was set at 1). *p<0.05, **p<0.005.

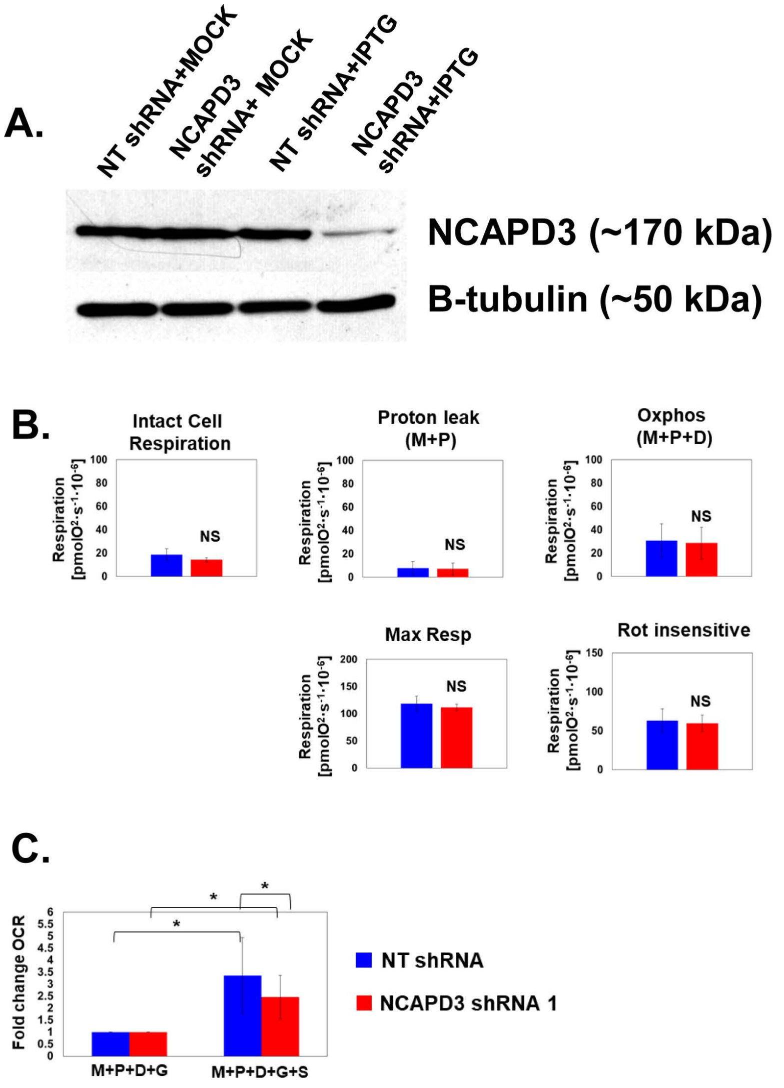

Figure S5. NCAPD3 deficient primary cells exhibit defects in mitochondrial Complex II function. A) ARPE-19 cells were treated with PBS (Mock) or IPTG to induce expression of Non-Target (NT) shRNA or NCAPD3 shRNA 1. Cell lysates were immunoblotted for NCAPD3 protein or β-tubulin as a loading control. NCAPD3 levels in NCAPD3 shRNA expressing cells were compared to levels in Non-Target shRNA expressing cells, which were set to 100%. B) High resolution respirometry was performed to measure oxygen consumption rates in intact non-permeabilized ARPE-19 cells in mitochondrial respiration buffer, using the Oroboros instrument. Cells were permeabilized with digitonin and electron transport chain complex-specific substrates

Journal of Cell Science • Supplementary information

and inhibitors were added sequentially, as described in methods (95,96). Proton leak and oxidative phosphorylation (OXPHOS) in response to Complex I-III substrates malate (M), pyruvate (P), ADP (D), glutamate (G) and Complex II substrate, succinate (S) were measured. Complex II activity was determined by subtracting the oxygen consumption rates following addition of M,P, and G from the oxygen consumption rates following addition of M,P,G, and S. Maximum respiration (Max R) as well as reserve respiratory (RR) capacity (response to protonophore, FCCP) were quantified. Rotenone-sensitive and –insensitive respiration, followed by complex IV function were also measured. Experiments were performed in ARPE-19 cells induced to express NCAPD3 shRNA 1 (red bars) or NT shRNA (blue bars). Four biological replicates were performed for each cell line and significance was determined using paired t-tests. C) Fold change in oxygen consumption rates (OCR) were measured in the ARPE-19 cells described above by normalizing the OCR following addition of succinate to the OCR following addition of glutamate (which was set at 1). *p<0.05, **p<0.005.

A.

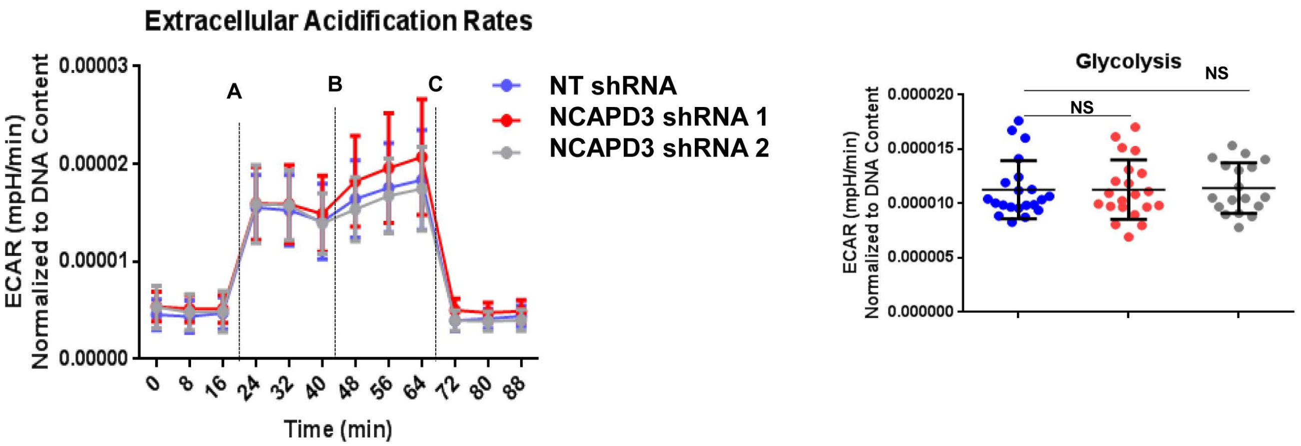

B.

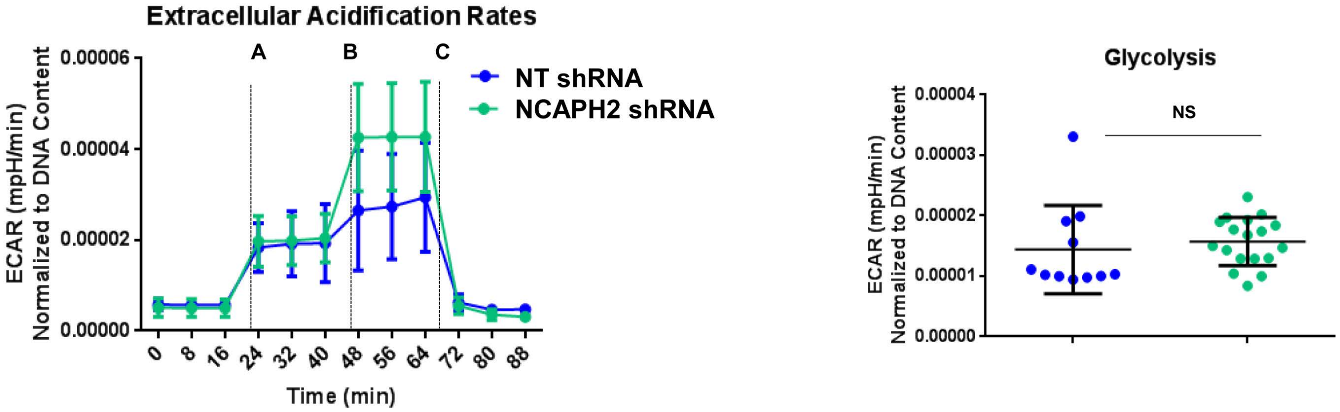

Figure S6. Loss of NCAPD3/ NCAPH2 does not impact glycolysis. Seahorse Glycolysis Stress Test assay in (A) Non-Target (n=21) and CAP-D3 (n=21, n=18) shRNA expressing cells (3 experimental replicates) and (B) Non-Target (n=11) and NCAPH2 (n=18) shRNA expressing cells (2 experimental replicates). Cells were injected with compounds at the time points indicated A- Glucose, B- Oligomycim, C- 2-DG. Graphs illustrate the full extracellular acidification rate profile (ECAR), rate of glycolysis, glycolytic capacity, glycolytic reserve, and non-glycolytic acidification rate. The first graphs in each panel illustrates the full extracellular acidification rate profile,

Journal of Cell Science • Supplementary information

showing extracellular acidification rates (y-axis) over time (x-axis). The second graph shows the rate of glycolysis which was calculated by subtracting the basal ECAR values from ECAR values generation upon stimulation with glucose. All values are normalized to DNA content by measuring the A485/A535 absorbance ratio using the Cyquant Cell Proliferation assay. A student t-test was used to determine significance.

Journal of Cell Science • Supplementary information

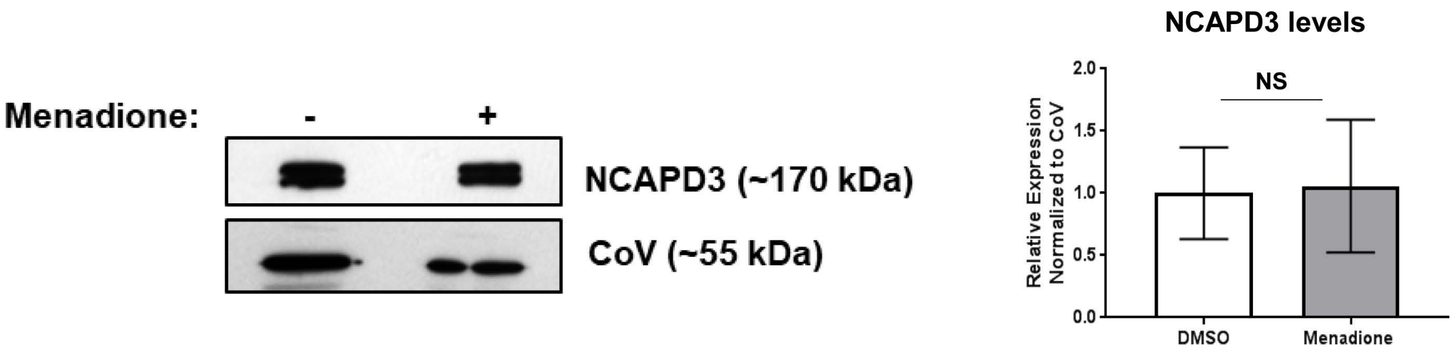

Figure S7. Menadione treatment does not affect mitochondrial NCAPD3 levels. Immunoblot analyses were performed to detect NCAPD3 protein levels present in mitochondrial lysates isolated from DMSO and menadione treated cells. CoV served as a loading control. Bar graphs show average quantification of NCAPD3 levels from two independent experiments. A student t-test was used to determine significance.

Journal of Cell Science • Supplementary information

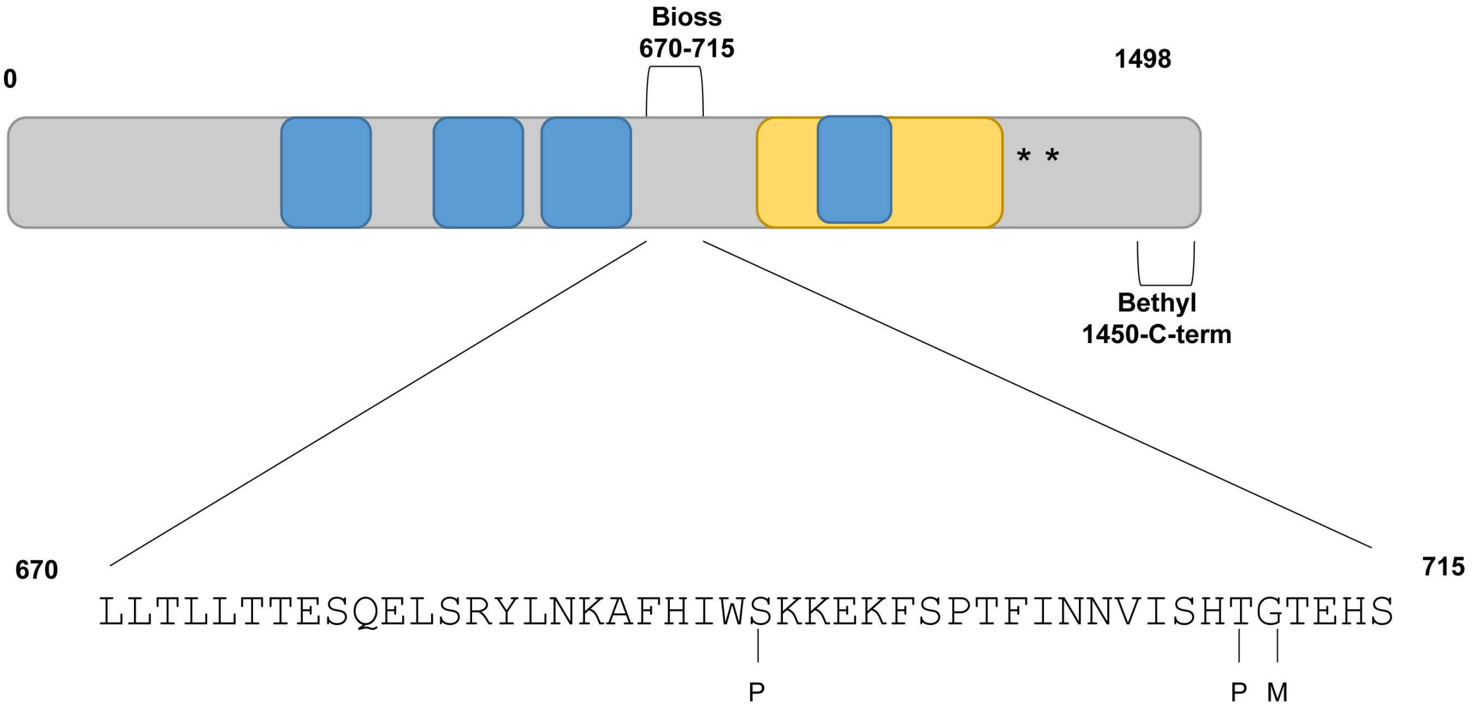

Figure S8. Predicted post translational modification sites to NCAPD3. Schematic of NCAPD3 sequence showing the amino acid residues with predicted modifications based on ExPASy Prosite analysis in the region recognized by the Bioss NCAPD3 antibody. P represents predicted phosphorylation sites. M represents predicted myristylation sites.

Journal of Cell Science • Supplementary information

Table S1. List of deregulated genes comparing DMSO treated Non-Target and NCAPD3 shRNA-1 expressing cells. $\log_2$ fold change > 1, $p < 0.05$.

Click here to Download Table S1

Table S2. List of MitoCarta-associated deregulated genes comparing DMSO treated Non-Target and NCAPD3 shRNA-1 expressing cells. $\log_2$ fold change > 1, $p < 0.05$.

Click here to Download Table S2

Table S3. List of deregulated genes comparing DMSO treated Non-Target and NCAPH2 shRNA-1 expressing cells. $\log_2$ fold change > 1, $p < 0.05$.

Click here to Download Table S3

Journal of Cell Science • Supplementary information

Table S4. Gene Ontology analysis for genes deregulated following NCAPH2 depletion.

Click here to Download Table S4

Table S5. List of MitoCarta-associated deregulated genes comparing DMSO treated Non-Target and NCAPH2 shRNA expressing cells. log_2 fold change > 1, $p < 0.05$.

Click here to Download Table S5

Table S6. List of deregulated genes in Non-Target and NCAPD3 shRNA expressing cells after menadione treatment. log_2 fold change > 1, $p < 0.05$.

Click here to Download Table S6

Journal of Cell Science • Supplementary information
